# Supplementary material for: Opioid-related treatment, interventions, and outcomes among incarcerated persons: A systematic review
Source: PLoS Med. 2019 Dec 31;16(12):e1003002. doi: 10.1371/journal.pmed.1003002 (PMC6938347; doi:10.1371/journal.pmed.1003002)
Supplement: S1 List of Manuscripts Excluded — (DOCX) [file pmed.1003002.s001.docx]

**S1. List of full text manuscripts evaluated and excluded (See PRISMA Flowchart)**

1. Altice FL, Azbel L, Stone J, Brooks-Pollock E, Smyrnov P, Dvoriak S, Taxman FS, El-Bassel N, Martin NK, Booth R, Stöver H, Dolan K, Vickerman P. The perfect storm: incarceration and the high-risk environment perpetuating transmission of HIV, hepatitis C virus, and tuberculosis in Eastern Europe and Central Asia. Lancet. 2016 Sep 17;388(10050):1228-48.
2. Azbel L, Polonsky M, Wegman M, Shumskaya N, Kurmanalieva A, Asanov A, Wickersham JA, Dvoriak S, Altice FL. Intersecting epidemics of HIV, HCV, and syphilis among soon-to-be released prisoners in Kyrgyzstan: Implications for prevention and treatment. Int J Drug Policy. 2016 Nov;37:9-20.
3. Azbel L, Rozanova J, Michels I, Altice FL, Stöver H. A qualitative assessment of an abstinence-oriented therapeutic community for prisoners with substance use disorders in Kyrgyzstan. Harm Reduct J. 2017 Jul 10;14(1):43.
4. Azbel L, Wegman MP, Polonsky M, Bachireddy C, Meyer J, Shumskaya N, Kurmanalieva A, Dvoryak S, Altice FL. Drug injection within prison in Kyrgyzstan: elevated HIV risk and implications for scaling up opioid agonist treatments. Int J Prison Health. 2018 Sep 10;14(3):175-187.
5. Azbel L, Wickersham JA, Grishaev Y, Dvoryak S, Altice FL. Correlates of HIV infection and being unaware of HIV status among soon-to-be-released Ukrainian prisoners. J Int AIDS Soc. 2014 Sep 10;17:19005.
6. Azbel L, Wickersham JA, Wegman MP, Polonsky M, Suleymanov M, Ismayilov R, Dvoryak S, Rotberga S, Altice FL. Burden of substance use disorders, mental illness, and correlates of infectious diseases among soon-to-be released prisoners in Azerbaijan. Drug Alcohol Depend. 2015 Jun 1;151:68-75.
7. Bachireddy C, Bazazi AR, Kavasery R, Govindasamy S, Kamarulzaman A, Altice FL. Attitudes toward opioid substitution therapy and pre-incarceration HIV transmission behaviors among HIV-infected prisoners in Malaysia: implications for secondary prevention. Drug Alcohol Depend. 2011 Jul 1;116(1-3):151-7.
8. Besson J, Grivel J, Tomei A, Zullino D, Thorens G, Castro E, Hachaichi M, Devaud Cornaz C, Dudzus M, Gothuey I. [Addictions]. Rev Med Suisse. 2017 Jan 11;13(544-545):12-15.
9. Best D, Walkerb D, Astona E, Pegramc C and O’Donnell G. Assessing the impact of a high-intensity partnership between the police and drug treatment service in addressing the offending of problematic drug users. Policing & Society. 2010; 20(3):358-369
10. Beyrer C, Malinowska-Sempruch K, Kamarulzaman A, Kazatchkine M, Sidibe M, Strathdee SA. Time to act: a call for comprehensive responses to HIV in people who use drugs. Lancet. 2010 Aug 14;376(9740):551-63.
11. Bielen R, Stumo SR, Halford R, Werling K, Reic T, Stöver H, Robaeys G, Lazarus JV. Harm reduction and viral hepatitis C in European prisons: a cross-sectional survey of 25 countries. Harm Reduct J. 2018 May 11;15(1):25.
12. Bi-Mohammed Z, Wright NM, Hearty P, King N, Gavin H. Prescription opioid abuse in prison settings: A systematic review of prevalence, practice and treatment responses. Drug Alcohol Depend. 2017 Feb 1;171:122-131.
13. Borquez A, Beletsky L, Nosyk B, Strathdee SA, Madrazo A, Abramovitz D, Rafful C, Morales M, Cepeda J, Panagiotoglou D, Krebs E, Vickerman P, Claude Boily M, Thomson N, Martin NK. The effect of public health-oriented drug law reform on HIV incidence in people who inject drugs in Tijuana, Mexico: an epidemic modelling study. Lancet Public Health. 2018 Sep;3(9):e429-e437.
14. Bruce RD, Schleifer RA. Ethical and human rights imperatives to ensure medication-assisted treatment for opioid dependence in prisons and pre-trial detention. Int J Drug Policy. 2008 Feb;19(1):17-23.
15. Chakrapani V, Kamei R, Kipgen H, Kh JK. Access to harm reduction and HIV-related treatment services inside Indian prisons: experiences of formerly incarcerated injecting drug users. Int J Prison Health. 2013;9(2):82-91.
16. Conner BT1, Hampton AS, Hunter J, Urada D. Treating opioid use under California's Proposition 36: differential outcomes by treatment modality. J Psychoactive Drugs. 2011 Sep;Suppl 7:77-83.
17. Coviello DM, Cornish JW, Lynch KG, Alterman AI, O'Brien CP. A randomized trial of oral naltrexone for treating opioid-dependent offenders. Am J Addict. 2010 ;19(5):422-32.
18. Coviello DM, Cornish JW, Lynch KG, Boney TY, Clark CA, Lee JD, Friedmann PD, Nunes EV, Kinlock TW, Gordon MS, Schwartz RP, Nuwayser ES, O'Brien CP. A multisite pilot study of extended-release injectable naltrexone treatment for previously opioid-dependent parolees and probationers. Subst Abus. 2012;33(1):48-59.
19. Crits-Christoph P, Lundy C, Stringer M, Gallop R, Gastfriend DR. Extended-Release Naltrexone for Alcohol and Opioid Problems in Missouri Parolees and Probationers. J Subst Abuse Treat. 2015;56:54-60.
20. Cropsey KL, Lane PS, Hale GJ, Jackson DO, Clark CB, Ingersoll KS, Islam MA, Stitzer ML. Results of a pilot randomized controlled trial of buprenorphine for opioid dependent women in the criminal justice system. Drug Alcohol Depend. 2011;119(3):172-8
21. Cropsey KL, Lane PS, Perkins AC, Clark CB, Hardy S, McCullumsmith C, Stitzer ML. Buprenorphine and medication management in a community corrections population: a pilot study. J Addict Med. 2013;7(3):210-5
22. Crowley D, Cullen W, Laird E, Lambert JS, Mc Hugh T, Murphy C, Van Hout MC. Exploring Patient Characteristics and Barriers to Hepatitis C Treatment in Patients on Opioid Substitution Treatment Attending a Community Based Fibro-scanning Clinic. J Transl Int Med. 2017 Jun 30;5(2):112-119.
23. Culbert GJ, Pillai V, Bick J, Al-Darraji HA, Wickersham JA, Wegman MP, Bazazi AR, Ferro E, Copenhaver M, Kamarulzaman A, Altice FL. Confronting the HIV, Tuberculosis, Addiction, and Incarceration Syndemic in Southeast Asia: Lessons Learned from Malaysia. J Neuroimmune Pharmacol. 2016 Sep;11(3):446-55.
24. Culbert GJ, Waluyo A, Iriyanti M, Muchransyah AP, Kamarulzaman A, Altice FL. Within-prison drug injection among HIV-infected male prisoners in Indonesia: a highly constrained choice. Drug Alcohol Depend. 2015 Apr 1;149:71-9.
25. Culbert GJ, Waluyo A, Wang M, Putri TA, Bazazi AR, Altice FL. Adherence to Antiretroviral Therapy Among Incarcerated Persons with HIV: Associations with Methadone and Perceived Safety. AIDS Behav. 2019 Aug;23(8):2048-2058.
26. Cunningham EB, Hajarizadeh B, Amin J, Bretana N, Dore GJ, Degenhardt L, Larney S, Luciani F, Lloyd AR, Grebely J; HITS-p Investigators. Longitudinal injecting risk behaviours among people with a history of injecting drug use in an Australian prison setting: The HITS-p study. Int J Drug Policy. 2018 Apr;54:18-25.
27. Dalsbø TK, Steiro A, Strømme H, Reinar LM. Effectiveness of Tapering from Methadone or Buprenorphine Maintenance Treatment Compared to Traditional Maintenance Treatment for People with Opiate Addiction: Systematic Review. Oslo, Norway: Knowledge Centre for the Health Services at The Norwegian Institute of Public Health (NIPH); 2017 Mar 2. Available from <http://www.ncbi.nlm.nih.gov/books/NBK482101/>
28. Davstad I, Stenbacka M, Leifman A, Romelsjö A. An 18-year follow-up of patients admitted to methadone treatment for the first time. J Addict Dis. 2009;28(1):39-52.
29. de Andrade D, Ritchie J, Rowlands M, Mann E, Hides L. Substance Use and Recidivism Outcomes for Prison-Based Drug and Alcohol Interventions. Epidemiol Rev. 2018 Jun 1;40(1):121-133.
30. de la Fuente L, Bravo MJ, Jiménez-Mejías E, Sordo L, Pulido J, Barrio G. [Evolution of the need and coverage of opioid substitution treatments and needle exchange programmes in Spanish prisons, 1992-2009]. Rev Esp Sanid Penit. 2012;14(2):67-77.
31. DeBeck K, Kerr T, Li K, Milloy MJ, Montaner J, Wood E. Incarceration and drug use patterns among a cohort of injection drug users. Addiction. 2009 Jan;104(1):69-76.
32. Desmarais SL, Gray JS, Rade CB, Cohn AM, Doherty S, Knight K. Medication-Assisted Treatment and Violent Outcomes in Community-Based Offenders with Alcohol and Drug Use Problems. Psychol Violence. 2016;6(3):378-389.
33. Dolan K, Rodas A, Bode A. Drug and alcohol use and treatment for Australian Indigenous and non-Indigenous prisoners: demand reduction strategies. Int J Prison Health. 2015;11(1):30-8.
34. Dolan K, Teutsch S, Scheuer N, Levy M, Rawlinson W, Kaldor J, Lloyd A, Haber P. Incidence and risk for acute hepatitis C infection during imprisonment in Australia. Eur J Epidemiol. 2010 Feb;25(2):143-8.
35. Dolan K, Wirtz AL, Moazen B, Ndeffo-Mbah M, Galvani A, Kinner SA, Courtney R, McKee M, Amon JJ, Maher L, Hellard M, Beyrer C, Altice FL. Global burden of HIV, viral hepatitis, and tuberculosis in prisoners and detainees. Lancet. 2016 Sep 10;388(10049):1089-1102.
36. Epperson MW, Khan MR, El-Bassel N, Wu E, Gilbert L. A longitudinal study of incarceration and HIV risk among methadone maintained men and their primary female partners. AIDS Behav. 2011 Feb;15(2):347-55.
37. Fairbairn N, Hayashi K, Ti L, Kaplan K, Suwannawong P, Wood E, Kerr T. Compulsory drug detention and injection drug use cessation and relapse in Bangkok, Thailand. Drug Alcohol Rev. 2015 Jan;34(1):74-81.
38. Falade-Nwulia O, Irvin R, Merkow A, Sulkowski M, Niculescu A, Olsen Y, Stoller K, Thomas DL, Latkin C, Mehta SH. Barriers and facilitators of hepatitis C treatment uptake among people who inject drugs enrolled in opioid treatment programs in Baltimore. J Subst Abuse Treat. 2019 May;100:45-51.
39. Farnia M, Ebrahimi B, Shams A, Zamani S. Scaling up methadone maintenance treatment for opioid-dependent prisoners in Iran. Int J Drug Policy. 2010 Sep;21(5):422-4.
40. Fiscella K, Wakeman SE, Beletsky L. Implementing Opioid Agonist Treatment in Correctional Facilities. JAMA Intern Med. 2018 Sep 1;178(9):1153-1154.
41. Fotiou A, Kanavou E, Antaraki A, Richardson C, Terzidou M, Kokkevi A; Drug Related Infectious Diseases (DRID) Medical Doctors Group of OKANA. HCV/HIV coinfection among people who inject drugs and enter opioid substitution treatment in Greece: prevalence and correlates. Hepatol Med Policy. 2016 Aug 25;1:9.
42. Fresquez-Chavez KR, Fogger S. Reduction of opiate withdrawal symptoms with use of clonidine in a county jail. J Correct Health Care. 2015 Jan;21(1):27-34.
43. Friedmann PD, Green TC, Taxman FS, Harrington M, Rhodes AG, Katz E, O'Connell D, Martin SS, Frisman LK, Litt M, Burdon W, Clarke JG, Fletcher BW; Step'n Out Research Group of CJ-DATS. Collaborative behavioral management among parolees: drug use, crime and re-arrest in the Step'n Out randomized trial. Addiction. 2012;107(6):1099-108.
44. Garfinkle J, Andermann F, Shevell MI. Neurolathyrism in vapniarka: medical heroism in a concentration Camp. Can J Neurol Sci. 2011 Nov;38(6):839-44.
45. Garnick DW, Horgan CM, Acevedo A, Lee MT, Panas L, Ritter GA, Dunigan R, Bidorini A, Campbell K, Haberlin K, Huber A, Lambert-Wacey D, Leeper T, Reynolds M, Wright D. Criminal justice outcomes after engagement in outpatient substance abuse treatment. J Subst Abuse Treat. 2014;46(3):295-305
46. Gelpí-Acosta C, Guarino H, Benoit E, Deren S, Pouget ER, Rodríguez A. Injection risk norms and practices among migrant Puerto Rican people who inject drugs in New York City: The limits of acculturation theory. Int J Drug Policy. 2019 Jul;69:60-69.
47. Gordon MS, Kinlock TW, Schwartz RP, Couvillion KA, Sudec LJ, O'Grady KE, Vocci FJ, Shabazz H. Buprenorphine Treatment for Probationers and Parolees. Subst Abus. 2015;36(2):217-25.
48. Groot E, Kouyoumdjian FG, Kiefer L, Madadi P, Gross J, Prevost B, Jhirad R, Huyer D, Snowdon V, Persaud N. Drug Toxicity Deaths after Release from Incarceration in Ontario, 2006-2013: Review of Coroner's Cases. PLoS One. 2016 Jul 6;11(7):e0157512.
49. Gryczynski J, Kinlock TW, Kelly SM, O'Grady KE, Gordon MS, Schwartz RP. Opioid agonist maintenance for probationers: patient-level predictors of treatment retention, drug use, and crime. Subst Abus. 2012;33(1):30-9
50. Guydish J, Chan M, Bostrom A, Jessup M, Davis T, Marsh C. A Randomized Trial of Probation Case Management for Drug-Involved Women Offenders. Crime Delinq. 2011;57(2):167-198.
51. Handanagic S, Bozicevic I, Civljak M, Dominkovic Z, Sevic S, Barbaric J, Nemeth Blazic T, Dakovic Rode O, Begovac J. HIV and hepatitis C prevalence, and related risk behaviours among people who inject drugs in three cities in Croatia: Findings from respondent-driven sampling surveys. Int J Drug Policy. 2016 Jun;32:57-63.
52. Harcouët L. [Hospital pharmaceutical practice in prison]. Ann Pharm Fr. 2010 Sep;68(5):286-90.
53. Hayashi K, Ti L, Ayutthaya PPN, Suwannawong P, Kaplan K, Small W, Kerr T. Barriers to retention in methadone maintenance therapy among people who inject drugs in Bangkok, Thailand: a mixed-methods study. Harm Reduct J. 2017 Sep 7;14(1):63.
54. Hedrich D, Alves P, Farrell M, Stöver H, Møller L, Mayet S. The effectiveness of opioid maintenance treatment in prison settings: a systematic review.
55. Hedrich D, Farrell M. Opioid maintenance in European prisons: is the treatment gap closing? Addiction. 2012 Mar;107(3):461-3.
56. Herce ME, Muyoyeta M, Topp SM, Henostroza G, Reid SE. Coordinating the prevention, treatment, and care continuum for HIV-associated tuberculosis in prisons: a health systems strengthening approach. Curr Opin HIV AIDS. 2018 Nov;13(6):492-500.
57. Horton M, McDonald R, Green TC, Nielsen S, Strang J, Degenhardt L, Larney S. A mapping review of take-home naloxone for people released from correctional settings. Int J Drug Policy. 2017 Aug;46:7-16.
58. Huang YF, Kuo HS, Lew-Ting CY, Tian F, Yang CH, Tsai TI, Gange SJ, Nelson KE. Mortality among a cohort of drug users after their release from prison: an evaluation of the effectiveness of a harm reduction program in Taiwan. Addiction. 2011 Aug;106(8):1437-45.
59. Izenberg JM, Bachireddy C, Soule M, Kiriazova T, Dvoryak S, Altice FL. High rates of police detention among recently released HIV-infected prisoners in Ukraine: implications for health outcomes. Drug Alcohol Depend. 2013 Nov 1;133(1):154-60.
60. Izenberg JM, Bachireddy C, Wickersham JA, Soule M, Kiriazova T, Dvoriak S, Altice FL. Within-prison drug injection among HIV-infected Ukrainian prisoners: prevalence and correlates of an extremely high-risk behaviour. Int J Drug Policy. 2014 Sep;25(5):845-52.
61. Joudrey PJ, Khan MR, Wang EA, Scheidell JD, Edelman EJ, McInnes DK, Fox AD. A conceptual model for understanding post-release opioid-related overdose risk. Addict Sci Clin Pract. 2019 Apr 15;14(1):17.
62. Jürgens R, Ball A, Verster A. Interventions to reduce HIV transmission related to injecting drug use in prison. Lancet Infect Dis. 2009 Jan;9(1):57-66.
63. Jürgens R, Nowak M, Day M. HIV and incarceration: prisons and detention. J Int AIDS Soc. 2011 May 19;14:26. doi: 10.1186/1758-2652-14-26.
64. Kamarulzaman A, Reid SE, Schwitters A, Wiessing L, El-Bassel N, Dolan K, Moazen B, Wirtz AL, Verster A, Altice FL. Prevention of transmission of HIV, hepatitis B virus, hepatitis C virus, and tuberculosis in prisoners. Lancet. 2016 Sep 10;388(10049):1115-1126.
65. Kamarulzaman A, Verster A, Altice FL. Prisons: ignore them at our peril. Curr Opin HIV AIDS. 2019 Sep;14(5):415-422.
66. Kelly SM, Oʼgrady KE, Jaffe JH, Gandhi D, Schwartz RP. Improvements in outcomes in methadone patients on probation/parole regardless of counseling early in treatment. J Addict Med. 2013;7(2):133-8.
67. Kimber J, Copeland L, Hickman M, Macleod J, McKenzie J, De Angelis D, Robertson JR. Survival and cessation in injecting drug users: prospective observational study of outcomes and effect of opiate substitution treatment. BMJ. 2010 Jul 1;341:c3172.
68. Kobayashi L1, Green TC, Bowman SE, Ray MC, McKenzie MS, Rich JD. Patient Simulation for Assessment of Layperson Management of Opioid Overdose With Intranasal Naloxone in a Recently Released Prisoner Cohort. Simul Healthc. 2017;12(1):22-27.
69. Kontautaite A, Matyushina-Ocheret D, Plotko M, Golichenko M, Kalvet M, Antonova L. Study of human rights violations faced by women who use drugs in Estonia. Harm Reduct J. 2018 Nov 6;15(1):54.
70. Krebs E, Min JE, Evans E, Li L, Liu L, Huang D, Urada D, Kerr T, Hser YI, Nosyk B. Estimating State Transitions for Opioid Use Disorders. Med Decis Making. 2017 Jul;37(5):483-497.
71. Krebs E, Urada D, Evans E, Huang D, Hser YI, Nosyk B. The costs of crime during and after publicly funded treatment for opioid use disorders: a population-level study for the state of California. Addiction. 2017 May;112(5):838-851.
72. Kunøe N, Lobmaier P, Ngo H, Hulse G. Injectable and implantable sustained release naltrexone in the treatment of opioid addiction. Br J Clin Pharmacol. 2014 Feb;77(2):264-71.
73. Laqueille X, Launay C, Dervaux A, Kanit M. [Abuse of alcohol and benzodiazepine during substitution therapy in heroin addicts: a review of the literature]. Encephale. 2009 Jun;35(3):220-5.
74. Larney S, Dolan K. A literature review of international implementation of opioid substitution treatment in prisons: equivalence of care? Eur Addict Res. 2009;15(2):107-12.
75. Larney S, Grebely J, Hickman M, De Angelis D, Dore GJ, Degenhardt L. Defining populations and injecting parameters among people who inject drugs: Implications for the assessment of hepatitis C treatment programs. Int J Drug Policy. 2015 Oct;26(10):950-7.
76. Larney S. Does opioid substitution treatment in prisons reduce injecting-related HIV risk behaviours? A systematic review. Addiction. 2010 Feb;105(2):216-23.
77. Leach D, Oliver P. Drug-related death following release from prison: a brief review of the literature with recommendations for practice. Curr Drug Abuse Rev. 2011 Dec;4(4):292-7.
78. Lee JD, Rich JD. Opioid pharmacotherapy in criminal justice settings: now is the time. Subst Abus. 2012;33(1):1-4.
79. Luciani F, Bretaña NA, Teutsch S, Amin J, Topp L, Dore GJ, Maher L, Dolan K, Lloyd AR; HITS-p investigators. A prospective study of hepatitis C incidence in Australian prisoners. Addiction. 2014 Oct;109(10):1695-706.
80. Ludwig AS, Peters RH. Medication-assisted treatment for opioid use disorders in correctional settings: an ethics review. Int J Drug Policy. 2014 Nov;25(6):1041-6.
81. Marco A, Gallego C, Caylà JA. Incidence of hepatitis C infection among prisoners by routine laboratory values during a 20-year period. PLoS One. 2014 Feb 28;9(2):e90560.
82. Marco A, López-Burgos A, García-Marcos L, Gallego C, Antón JJ, Errasti A. [Is the availability of buprenorphine/naloxone therapy for opioid-dependent inmates a necessity? ]. Rev Esp Sanid Penit. 2013 Feb;15(3):105-13.
83. Maru DS, Bruce RD, Basu S, Altice FL. Clinical outcomes of hepatitis C treatment in a prison setting: feasibility and effectiveness for challenging treatment populations. Clin Infect Dis. 2008 Oct 1;47(7):952-61.
84. Mazhnaya A, Bojko MJ, Marcus R, Filippovych S, Islam Z, Dvoriak S, Altice FL. In Their Own Voices: Breaking the Vicious Cycle of Addiction, Treatment and Criminal Justice Among People who Inject Drugs in Ukraine. Drugs (Abingdon Engl). 2016;23(2):163-175.
85. Meng J, Burris S. The role of the Chinese police in methadone maintenance therapy: a literature review. Int J Drug Policy. 2013 Nov;24(6):e25-34.
86. Merrall EL, Kariminia A, Binswanger IA, Hobbs MS, Farrell M, Marsden J, Hutchinson SJ, Bird SM. Meta-analysis of drug-related deaths soon after release from prison. Addiction. 2010 Sep;105(9):1545-54.
87. Michel L, Jauffret-Roustide M, Blanche J, Maguet O, Calderon C, Cohen J, Carrieri PM; ANRS PRI²DE study group. Limited access to HIV prevention in French prisons (ANRS PRI2DE): implications for public health and drug policy. BMC Public Health. 2011 May 27;11:400.
88. Milloy MJ, Wood E. Withdrawal from methadone in US prisons: cruel and unusual? Lancet. 2015 Jul 25;386(9991):316-8.
89. Mistral W, Wilkinson S, Mastache C, Midgley S, Law F. Efficacy of naltrexone treatment with combined crack and opiate users: A descriptive study of a new treatment service in Bristol, UK. Drugs Educ Prev Pol, 2008; 15(1): 107–119
90. Mitchell SG, Gryczynski J, Kelly SM, O'Grady KE, Jaffe JH, Olsen YK, Schwartz RP. Treatment Outcomes of African American Buprenorphine Patients by Parole and Probation Status. J Drug Issues. 2014 Jan;44(1):69-82.
91. Mohammed Z, Hughes GJ, Hearty P, Wright NMJ. The perceived and actual consequences of intranasal administration of buprenorphine or burprenorphine–naloxone by prisoners. Drugs Educ Prev Pol. 2016; 23(2): 99–102
92. Moller LF, van den Bergh BJ, Karymbaeva S, Esenamanova A, Muratalieva R. Drug use in prisons in Kyrgyzstan: a study about the effect of health promotion among prisoners. Int J Prison Health. 2008;4(3):124-33.
93. Moore KE, Roberts W, Reid HH, Smith KMZ, Oberleitner LMS, McKee SA. Effectiveness of medication assisted treatment for opioid use in prison and jail settings: A meta-analysis and systematic review. J Subst Abuse Treat. 2019 Apr;99:32-43.
94. Moradi G, Farnia M, Shokoohi M, Shahbazi M, Moazen B, Rahmani K. Methadone maintenance treatment program in prisons from the perspective of medical and non-medical prison staff: a qualitative study in Iran. Int J Health Policy Manag. 2015 Mar 12;4(9):583-9.
95. Morozova O, Azbel L, Grishaev Y, Dvoryak S, Wickersham JA, Altice FL. Ukrainian prisoners and community reentry challenges: implications for transitional care. Int J Prison Health. 2013;9(1):5-19.
96. Mukherjee TI, Wickersham JA, Desai MM, Pillai V, Kamarulzaman A, Altice FL. Factors associated with interest in receiving prison-based methadone maintenance therapy in Malaysia. Drug Alcohol Depend. 2016 Jul 1;164:120-127.
97. Müller J, Schmidt D, Kollan C, Lehmann M, Bremer V, Zimmermann R. High variability of TB, HIV, hepatitis C treatment and opioid substitution therapy among prisoners in Germany. BMC Public Health. 2017 Oct 25;17(1):843.
98. Navadeh S, Mirzazadeh A, Gouya MM, Farnia M, Alasvand R, Haghdoost AA. HIV prevalence and related risk behaviours among prisoners in Iran: results of the national biobehavioural survey, 2009. Sex Transm Infect. 2013 Nov;89 Suppl 3:iii33-6
99. Nunes EV, Gordon M, Friedmann PD, Fishman MJ, Lee JD, Chen DT, Hu MC, Boney TY, Wilson D, O'Brien CP. Relapse to opioid use disorder after inpatient treatment: Protective effect of injection naltrexone. J Subst Abuse Treat. 2018 Feb;85:49-55.
100. Opitz-Welke A, Lehmann M, Seidel P, Konrad N. Medicine in the Penal System. Dtsch Arztebl Int. 2018 Nov 30;115(48):808-814.
101. Papaluca T, McDonald L, Craigie A, Gibson A, Desmond P, Wong D, Winter R, Scott N, Howell J, Doyle J, Pedrana A, Lloyd A, Stoove M, Hellard M, Iser D, Thompson A. Outcomes of treatment for hepatitis C in prisoners using a nurse-led, statewide model of care. J Hepatol. 2019 May;70(5):839-846.
102. Pauly V, Frauger E, Rouby F, Sirere S, Monier S, Paulet C, Gibaga V, Micallef J, Thirion X. [Analysis of addictive behaviours among new prisoners in France using the OPPIDUM program]. Encephale. 2010 Apr;36(2):122-31.
103. Petterson AG, Madah-Amiri D. Overdose prevention training with naloxone distribution in a prison in Oslo, Norway: a preliminary study. Harm Reduct J. 2017 Nov 21;14(1):74.
104. Pierce M, Bird SM, Hickman M, Marsden J, Dunn G, Seddon T, Millar T. Effect of initiating drug treatment on the risk of drug-related poisoning death and acquisitive crime among offending heroin users. Int J Drug Policy. 2018 Jan;51:42-51.
105. Pierce M, Bird SM, Hickman M, Marsden J, Dunn G, Seddon T, Millar T. Effect of initiating drug treatment on the risk of drug-related poisoning death and acquisitive crime among offending heroin users. Int J Drug Policy. 2018 Jan;51:42-51.
106. Polonsky M, Azbel L, Wickersham JA, Marcus R, Doltu S, Grishaev E, Dvoryak S, Altice FL. Accessing methadone within Moldovan prisons: Prejudice and myths amplified by peers. Int J Drug Policy. 2016 Mar;29:91-5.
107. Polonsky M, Azbel L, Wickersham JA, Taxman FS, Grishaev E, Dvoryak S, Altice FL. Challenges to implementing opioid substitution therapy in Ukrainian prisons: Personnel attitudes toward addiction, treatment, and people with HIV/AIDS. Drug Alcohol Depend. 2015 Mar 1;148:47-55.
108. Polonsky M, Rozanova J, Azbel L, Bachireddy C, Izenberg J, Kiriazova T, Dvoryak S, Altice FL. Attitudes Toward Addiction, Methadone Treatment, and Recovery Among HIV-Infected Ukrainian Prisoners Who Inject Drugs: Incarceration Effects and Exploration of Mediators. AIDS Behav. 2016 Dec;20(12):2950-2960.
109. Rhodes T, Azbel L, Lancaster K, Meyer J. The becoming-methadone-body: on the onto-politics of health intervention translations. Sociol Health Illn. 2019 Jul 16.
110. Robertson AG, Easter MM, Lin HJ, Frisman LK, Swanson JW, Swartz MS. Associations between pharmacotherapy for opioid dependence and clinical and criminal justice outcomes among adults with co-occurring serious mental illness. J Subst Abuse Treat. 2018 Mar;86:17-25.
111. Rozanova J, Morozova O, Azbel L, Bachireddy C, Izenberg JM, Kiriazova T, Dvoryak S, Altice FL. Perceptions of Health-Related Community Reentry Challenges among Incarcerated Drug Users in Azerbaijan, Kyrgyzstan, and Ukraine. J Urban Health. 2018 Aug;95(4):508-522.
112. Rubenstein LS, Amon JJ, McLemore M, Eba P, Dolan K, Lines R, Beyrer C. HIV, prisoners, and human rights. Lancet. 2016 Sep 17;388(10050):1202-14.
113. Russolillo A, Moniruzzaman A, McCandless LC, Patterson M, Somers JM. Associations between methadone maintenance treatment and crime: a 17-year longitudinal cohort study of Canadian provincial offenders. Addiction. 2018 Apr;113(4):656-667.
114. Saber-Tehrani AS, Springer SA, Qiu J, Herme M, Wickersham J, Altice FL. Rationale, study design and sample characteristics of a randomized controlled trial of directly administered antiretroviral therapy for HIV-infected prisoners transitioning to the community - a potential conduit to improved HIV treatment outcomes. Contemp Clin Trials. 2012 Mar;33(2):436-44.
115. Sacks JY1, McKendrick K, Hamilton Z, Cleland CM, Pearson FS, Banks S.
116. Sander G, Murphy F. The furthest left behind: the urgent need to scale up harm reduction in prisons. Int J Prison Health. 2017 Sep 11;13(3-4):185-191.
117. Sander G, Scandurra A, Kamenska A, MacNamara C, Kalpaki C, Bessa CF, Laso GN, Parisi G, Varley L, Wolny M, Moudatsou M, Pontes NH, Mannix-McNamara P, Libianchi S, Antypas T. Overview of harm reduction in prisons in seven European countries. Harm Reduct J. 2016 Oct 7;13(1):28.
118. Sander G, Shirley-Beavan S, Stone K. The Global State of Harm Reduction in Prisons. J Correct Health Care. 2019 Apr;25(2):105-120.
119. Schwartz RP, Kelly SM, Mitchell SG, Dunlap L, Zarkin GA, Sharma A, O'Grady KE, Jaffe JH. Interim methadone and patient navigation in jail: Rationale and design of a randomized clinical trial. Contemp Clin Trials. 2016 Jul;49:21-8.
120. Shahbazi M, Farnia M, Moradi G, Karamati M, Paknazar F, Mirmohammad Khani M. Injecting Drug Users Retention in Needle-Exchange Program and its Determinants in Iran Prisons. Int J High Risk Behav Addict. 2015 Jun 20;4(2):e23751.
121. Sharma A, O'Grady KE, Kelly SM, Gryczynski J, Mitchell SG, Schwartz RP. Pharmacotherapy for opioid dependence in jails and prisons: research review update and future directions. Subst Abuse Rehabil. 2016 Apr 27;7:27-40.
122. Sheard L, Wright NM, Adams CE, Bound N, Rushforth B, Hart R, Tompkins CN. The Leeds Evaluation of Efficacy of Detoxification Study (LEEDS) Prisons Project Study: protocol for a randomised controlled trial comparing methadone and buprenorphine for opiate detoxification. Trials. 2009 Jul 14;10:53.
123. Shrestha R, Weikum D, Copenhaver M, Altice FL. The Influence of Neurocognitive Impairment, Depression, and Alcohol Use Disorders on Health-Related Quality of Life among Incarcerated, HIV-Infected, Opioid Dependent Malaysian Men: A Moderated Mediation Analysis. AIDS Behav. 2017 Apr;21(4):1070-1081.
124. Silbernagl M, Slamanig R, Fischer G, Brandt L. Hepatitis C infection and psychiatric burden in two imprisoned cohorts: Young offenders and opioid-maintained prisoners. Health Policy. 2018 Dec;122(12):1392-1402.
125. Smirnov A, Kemp R, Ward J, Henderson S, Williams S, Dev A, Najman JM. Hepatitis C viral infection and imprisonment among Aboriginal and Torres Strait Islander and non-Indigenous people who inject drugs. Drug Alcohol Rev. 2018 Nov;37(7):831-836.
126. Snow KJ, Young JT, Preen DB, Lennox NG, Kinner SA. Incidence and correlates of hepatitis C virus infection in a large cohort of prisoners who have injected drugs. BMC Public Health. 2014 Aug 11;14:830. doi: 10.1186/1471-2458-14-830.
127. Søholm J, Holm DK, Mössner B, Madsen LW, Hansen JF, Weis N, Sauer AP, Awad T, Christensen PB. Incidence, prevalence and risk factors for hepatitis C in Danish prisons. PLoS One. 2019 Jul 26;14(7):e0220297.
128. Sondhi A, Ryan G, Day E. Stakeholder perceptions and operational barriers in the training and distribution of take-home naloxone within prisons in England. Harm Reduct J. 2016 Feb 3;13:5.
129. Sondhi A. Addressing perceptions of opiate-using prisoners to take-home naloxone: findings from one English region. Drugs and Alcohol Today. 2016 ; 16(2): 124-130.
130. Springer SA, Bruce RD. A pilot survey of attitudes and knowledge about opioid substitution therapy for HIV-infected prisoners. J Opioid Manag. 2008 Mar-Apr;4(2):81-6.
131. Stone KA. Reviewing harm reduction for people who inject drugs in Asia: the necessity for growth. Harm Reduct J. 2015 Oct 16;12:32.
132. Stöver H, Meroueh F, Marco A, Keppler K, Saiz de la Hoya P, Littlewood R, Wright N, Nava F, Alam F, Walcher S, Somaini L. Offering HCV treatment to prisoners is an important opportunity: key principles based on policy and practice assessment in Europe. BMC Public Health. 2019 Jan 8;19(1):30.
133. Strang J, McDonald R, Campbell G, Degenhardt L, Nielsen S, Ritter A, Dale O. Take-Home Naloxone for the Emergency Interim Management of Opioid Overdose: The Public Health Application of an Emergency Medicine. Drugs. 2019 Sep;79(13):1395-1418.
134. Suntharasamai P, Martin M, Vanichseni S, van Griensven F, Mock PA, Pitisuttithum P, Tappero JW, Sangkum U, Kitayaporn D, Gurwith M, Choopanya K; Bangkok Vaccine Evaluation Group. Factors associated with incarceration and incident human immunodeficiency virus (HIV) infection among injection drug users participating in an HIV vaccine trial in Bangkok, Thailand, 1999-2003. Addiction. 2009 Feb;104(2):235-42.
135. Underhill K, Dumont D, Operario D. HIV prevention for adults with criminal justice involvement: a systematic review of HIV risk-reduction interventions in incarceration and community settings. Am J Public Health. 2014 Nov;104(11):e27-53.
136. Vagenas P, Azbel L, Polonsky M, Kerimi N, Mamyrov M, Dvoryak S, Altice FL. A review of medical and substance use co-morbidities in Central Asian prisons: implications for HIV prevention and treatment. Drug Alcohol Depend. 2013 Nov;132 Suppl 1:S25-31.
137. Wiessing L, Ferri M, Běláčková V, Carrieri P, Friedman SR, Folch C, Dolan K, Galvin B, Vickerman P, Lazarus JV, Mravčík V, Kretzschmar M, Sypsa V, Sarasa-Renedo A, Uusküla A, Paraskevis D, Mendão L, Rossi D, van Gelder N, Mitcheson L, Paoli L, Gomez CD, Milhet M, Dascalu N, Knight J, Hay G, Kalamara E, Simon R; EUBEST working group, Comiskey C, Rossi C, Griffiths P. Monitoring quality and coverage of harm reduction services for people who use drugs: a consensus study. Harm Reduct J. 2017 Apr 22;14(1):19.
138. Wolfe D, Carrieri MP, Shepard D. Treatment and care for injecting drug users with HIV infection: a review of barriers and ways forward. Lancet. 2010 Jul 31;376(9738):355-66.
139. Yen YF, Rodwell TC, Yen MY, Hsu YH, Chuang P, Li LH, Su LW, Yang YH, Jiang XR, Fang YC, Garfein RS. HIV infection risk among injection drug users in a methadone maintenance treatment program, Taipei, Taiwan 2007-2010. Am J Drug Alcohol Abuse. 2012 Nov;38(6):544-50.
140. Zamani S, Farnia M, Torknejad A, Alaei BA, Gholizadeh M, Kasraee F, Ono-Kihara M, Oba K, Kihara M. Patterns of drug use and HIV-related risk behaviors among incarcerated people in a prison in Iran. J Urban Health. 2010 Jul;87(4):603-16.
