# Supplementary material for: Opioid-related treatment, interventions, and outcomes among incarcerated persons: A systematic review
Source: PLoS Med. 2019 Dec 31;16(12):e1003002. doi: 10.1371/journal.pmed.1003002 (PMC6938347; doi:10.1371/journal.pmed.1003002)
Supplement: S1 MEDLINE Search Strategy — (DOCX) [file pmed.1003002.s002.docx]

**S1 MEDLINE Search Strategy**

Database: Ovid MEDLINE(R) <1946 to December Week 4 2018>, Ovid MEDLINE(R) In-Process & Other Non-Indexed Citations <December 31, 2018>, Ovid MEDLINE(R) Epub Ahead of Print <December 31, 2018>

Search Strategy:

--------------------------------------------------------------------------------

1 exp Opioid-Related Disorders/

2 opiate$.mp.

3 opiod$.mp.

4 opioid$.mp.

5 heroin$.mp.

6 exp Substance Abuse, Intravenous/

7 exp Opiate Alkaloids/

8 morphine$.mp.

9 oxycodone$.mp.

10 codeine$.mp.

11 fentanyl$.mp.

12 hydrocodone$.mp.

13 hydromorphone$.mp.

14 meperidine$.mp.

15 oxymorphone$.mp.

16 exp Needle Sharing/

17 ((intraven$ or inject$) adj3 abus$).mp.

18 ((intraven$ or inject$) adj3 us$).mp.

19 (needle$ adj3 shar$).mp.

20 (syring$ adj3 shar$).mp.

21 exp prison/

22 exp prisoners/

23 (criminal adj3 justice$).mp.

24 (correction$ adj3 system$).mp.

25 (correction$ adj3 facilit$).mp.

26 imprison$.mp.

27 detention$.mp.

28 jail$.mp.

29 prison$.mp.

30 bail$.mp.

31 parole$.mp.

32 probat$.mp.

33 offender$.mp.

34 felon$.mp.

35 (delinquen$ adj3 juvenile$).mp.

36 (justice adj3 juvenile$).mp.

37 (criminal$ adj3 justice$).mp.

38 exp Halfway Houses/

39 (halfway adj3 hous$).mp.

40 penitentiar$.mp.

41 postincarcerat$.mp.

42 post-incarcerat$.mp.

43 exp METHADONE/

44 exp Opiate Substitution Treatment/

45 exp Buprenorphine/

46 exp Naloxone/

47 exp Naltrexone/

48 buprenorphine$.mp.

49 methadone$.mp.

50 naltrexone$.mp.

51 naloxone$.mp.

52 diamorphine$.mp.

53 levacetylmethadol$.mp.

54 LAAM?.mp.

55 levomethadyl acetate$.mp.

56 ((substitut$ or maintain$ or mainten$ or replac$ or detox$) adj3 opi$).mp. (4860)

57 ((control$ or prevent$ or avert$ or reduc$ or minimiz$ or reduc$ or manag$) adj3 overdos$).mp.

58 exp Psychotherapy/

59 exp Mental Health Services/

60 exp Therapeutic Community/

61 exp Motivational Interviewing/

62 exp Behavior Therapy/

63 exp COUNSELING/

64 exp Self-Help Groups/

65 therap$.mp.

66 counsel?ing$.mp.

67 self$ help$.mp.

68 support$ group$.mp.

69 narcotic$ anonymous$.mp.

70 addict$ anonymous$.mp.

71 intervent$.mp.

72 treat$.mp.

73 service$.mp.

74 psychotherap$.mp.

75 (motivational adj3 interviewing$).mp.

76 therapeutic communit$.mp.

77 or/1-20 [opiate abuse set]

78 or/21-42 [criminal justice system set]

79 or/43-77 [intervention set]

80 78 and 79 and 80

81 exp animals/ not humans.sh.

82 81 not 82

83 limit 83 to yr="2008 -Current"
