# Supplementary material for: Opioid-related treatment, interventions, and outcomes among incarcerated persons: A systematic review
Source: PLoS Med. 2019 Dec 31;16(12):e1003002. doi: 10.1371/journal.pmed.1003002 (PMC6938347; doi:10.1371/journal.pmed.1003002)
Supplement: S1 Prospero Registration — (PDF) [file pmed.1003002.s004.pdf]

PROSPERO

International prospective register of systematic reviews

1. * Review title.

Give the working title of the review, for example the one used for obtaining funding. Ideally the title should state succinctly the interventions or exposures being reviewed and the associated health or social problems. Where appropriate, the title should use the PI(E)COS structure to contain information on the Participants, Intervention (or Exposure) and Comparison groups, the Outcomes to be measured and Study designs to be included.

Opioid-related treatment, interventions and outcomes among correctional populations: a systematic review

2. Original language title.

For reviews in languages other than English, this field should be used to enter the title in the language of the review. This will be displayed together with the English language title.

3. * Anticipated or actual start date.

Give the date when the systematic review commenced, or is expected to commence.

03/09/2018

4. * Anticipated completion date.

Give the date by which the review is expected to be completed.

29/11/2019

5. * Stage of review at time of this submission.

Indicate the stage of progress of the review by ticking the relevant Started and Completed boxes. Additional information may be added in the free text box provided.

Please note: Reviews that have progressed beyond the point of completing data extraction at the time of initial registration are not eligible for inclusion in PROSPERO. Should evidence of incorrect status and/or completion date being supplied at the time of submission come to light, the content of the PROSPERO record will be removed leaving only the title and named contact details and a statement that inaccuracies in the stage of the review date had been identified.

This field should be updated when any amendments are made to a published record and on completion and publication of the review. If this field was pre-populated from the initial screening questions then you are not able to edit it until the record is published.

The review has not yet started: No

Review stage	Started	Completed
Preliminary searches	Yes	No
Piloting of the study selection process	Yes	No
Formal screening of search results against eligibility criteria	Yes	No
Data extraction	No	No

Review stage	Started	Completed
Risk of bias (quality) assessment	No	No
Data analysis	No	No

Provide any other relevant information about the stage of the review here (e.g. Funded proposal, protocol not yet finalised).

6. * Named contact.

The named contact acts as the guarantor for the accuracy of the information presented in the register record.

Monica Malta

Email salutation (e.g. "Dr Smith" or "Joanne") for correspondence:
Dr Malta

7. * Named contact email.

Give the electronic mail address of the named contact.

monica.malta@camh.ca

8. Named contact address

PLEASE NOTE this information will be published in the PROSPERO record so please do not enter private information

Give the full postal address for the named contact.

33 Russell Street / Room RS 2035, Toronto, Ontario, M5S 2S1, Canada

9. Named contact phone number.

Give the telephone number for the named contact, including international dialling code.

2262686093

10. * Organisational affiliation of the review.

Full title of the organisational affiliations for this review and website address if available. This field may be completed as 'None' if the review is not affiliated to any organisation.

University of Toronto

Organisation web address:
https://www.psychiatry.utoronto.ca/

11. * Review team members and their organisational affiliations.

Give the title, first name, last name and the organisational affiliations of each member of the review team. Affiliation refers to groups or organisations to which review team members belong.

Dr Monica Malta. CAMH
Dr Benedikt Fischer. University of Toronto

12. * Funding sources/sponsors.

Give details of the individuals, organizations, groups or other legal entities who take responsibility for initiating, managing, sponsoring and/or financing the review. Include any unique identification numbers assigned to the review by the individuals or bodies listed.

Canadian Institutes of Health Research (CIHR), Ontario CRISM Node Team (grant #SMN-139150)

13. * Conflicts of interest.

List any conditions that could lead to actual or perceived undue influence on judgements concerning the main topic investigated in the review.

None

14. Collaborators.

Give the name and affiliation of any individuals or organisations who are working on the review but who are not listed as review team members.

Mrs Thepikaa Varatharajan. CAMH

Miss Cayley Russell. CAMH

15. * Review question.

State the question(s) to be addressed by the review, clearly and precisely. Review questions may be specific or broad. It may be appropriate to break very broad questions down into a series of related more specific questions. Questions may be framed or refined using PI(E)COS where relevant.

This search included studies addressing individuals who were: 1) opioid users or have been diagnosed with an opioid use disorder either before or during incarceration and 2) were currently incarcerated or recently released from prison (within 12 months post-release). Studies were excluded if participants were: 1) not opioid users; 2) using opioids for medical purposes; 3) released from prison for more than 12 months and 4) on probation/parole, involved in drug treatment courts or diversion programs. Only studies presenting opioid-related interventions (i.e. addiction treatment, relapse or overdose prevention) initiated either in-prison or within the 12-months post-release were included.

16. * Searches.

State the sources that will be searched. Give the search dates, and any restrictions (e.g. language or publication period). Do NOT enter the full search strategy (it may be provided as a link or attachment.)

The search was developed by a health science librarian (SB), and included studies published between January 2008 and December 2018. We searched the following databases: Criminal Justice Abstracts, EMBASE, MEDLINE, National Criminal Justice Reference Service (NCJRS), PsycINFO, Scopus, and Web of Science. The search strategy was initially developed in MEDLINE and combined relevant MeSH and keywords on prison/post-incarceration populations (i.e. jail$, prison$, offender$, probat$, felon$, detention$, imprison$, postincarcerat$) with opiate abuse (i.e. exp Opioid-Related Disorders/, opiate$, opioid$), and treatment interventions (i.e. exp Opiate Substitution Treatment/, buprenorphine$, methadone$, naltrexone$, naloxone$). Detailed search strategies were developed and revised for each database individually, based on the MEDLINE search strategy. The reference lists of studies selected for inclusion in this review were also searched for relevant research. To identify grey literature publications, targeted searching of preselected websites (i.e. government and other organizational websites) was also conducted. We also contacted authors to clarify data when necessary.

17. URL to search strategy.

Give a link to a published pdf/word document detailing either the search strategy or an example of a search strategy for a specific database if available (including the keywords that will be used in the search strategies), or upload your search strategy.

Do NOT provide links to your search results.

https://www.crd.york.ac.uk/PROSPEROFILES/135900_STRATEGY_20190517.pdf

Do not make this file publicly available until the review is complete

18. * Condition or domain being studied.

Give a short description of the disease, condition or healthcare domain being studied. This could include health and wellbeing outcomes.

We will identify all relevant observational studies and randomized controlled trials (RCTs) that evaluated interventions addressing opioid use disorder among correctional adult population.

19. * Participants/population.

Give summary criteria for the participants or populations being studied by the review. The preferred format includes details of both inclusion and exclusion criteria.

This search included studies addressing individuals who were: 1) opioid users or have been diagnosed with an opioid use disorder either before or during incarceration and 2) were currently incarcerated or recently released from prison (within 12 months post-release).

20. * Intervention(s), exposure(s).

Give full and clear descriptions or definitions of the nature of the interventions or the exposures to be reviewed.

All intervention addressing opioid use disorder among correctional adult population were included.

21. * Comparator(s)/control.

Where relevant, give details of the alternatives against which the main subject/topic of the review will be compared (e.g. another intervention or a non-exposed control group). The preferred format includes details of both inclusion and exclusion criteria.

No controls were included

22. * Types of study to be included.

Give details of the types of study (study designs) eligible for inclusion in the review. If there are no restrictions on the types of study design eligible for inclusion, or certain study types are excluded, this should be stated. The preferred format includes details of both inclusion and exclusion criteria.

Only observational studies and randomized controlled trials (RCTs) were included

23. Context.

Give summary details of the setting and other relevant characteristics which help define the inclusion or exclusion criteria.

This search will include studies addressing individuals who were: 1) opioid users or have been diagnosed with an opioid use disorder either before or during incarceration and 2) were currently incarcerated or recently released from prison (within 12 months post-release). Studies will be excluded if participants were: 1) not opioid users; 2) using opioids for medical purposes; 3) released from prison for more than 12 months and 4) on probation/parole, involved in drug treatment courts or diversion programs. Only studies presenting opioid-related interventions (i.e. addiction treatment, relapse or overdose prevention) initiated either in-prison or within the 12-months post-release will be included.
Any study reporting the following intervention related outcomes will be included: mortality (all causes and/or opioid-related), treatment outcomes (i.e. relapse, retention, withdrawal effects), and recidivism (i.e. re-incarceration and arrests). Studies presenting cost-effectiveness analysis, opioid-treatment impact on blood-borne and/or sexually transmitted infections, self-screening or individual's subjective experiences/feelings/attitudes towards opioid-treatment will be excluded. Original, peer-reviewed articles published in English are eligible for inclusion. Reviews, editorials, commentaries, case series/case reports, guidelines, editorials, unpublished manuscripts, dissertations, and government reports will be excluded.

24. * Main outcome(s).

Give the pre-specified main (most important) outcomes of the review, including details of how the outcome is defined and measured and when these measurement are made, if these are part of the review inclusion criteria.

Main outcome is mortality (all causes and/or opioid-related) after prison release.

Timing and effect measures

Not included

25. * Additional outcome(s).

List the pre-specified additional outcomes of the review, with a similar level of detail to that required for main outcomes. Where there are no additional outcomes please state 'None' or 'Not applicable' as appropriate to the review

Any study reporting the following intervention related outcomes will be included: mortality (all causes and/or opioid-related), treatment outcomes (i.e. relapse, retention, withdrawal effects), and recidivism (i.e. re-incarceration and arrests). Studies presenting cost-effectiveness analysis, opioid-treatment impact on blood-borne and/or sexually transmitted infections, self-screening or individual's subjective experiences/feelings/attitudes towards opioid-treatment will be excluded.

Timing and effect measures

Not included

26. * Data extraction (selection and coding).

Describe how studies will be selected for inclusion. State what data will be extracted or obtained. State how this will be done and recorded.

Two reviewers independently will carry out the study selection, evaluation, and data extraction. If consensus could not be reached among reviewers, a third reviewer will be involved.

27. * Risk of bias (quality) assessment.

Describe the method of assessing risk of bias or quality assessment. State which characteristics of the studies will be assessed and any formal risk of bias tools that will be used.

For non RCT studies, risk of bias will be assessed utilizing the Cochrane's ROBINS-I ("Risk Of Bias In Non-randomised Studies - of Interventions"). The following characteristic will be evaluated: pre intervention (bias due to confounding, bias in selection of study participants); at intervention (Bias in classification of interventions); and post-intervention (bias due to deviations from intended interventions, bias due to missing data, bias in measurement of outcomes, bias in selection of reported results).

For RCT studies, risk of bias will be assessed utilizing the Cochrane's RoB 2(risk-of-bias tool for randomized trials), published on 9 July, 2019 will be utilized. We will focus our evaluation on five risk of bias items: random sequence generation, allocation concealment, blinding of participants and personnel, blinding of outcome assessment and incomplete outcome data.

For each item, we compared the risk of bias assessment in terms of 'high', 'low' or 'unclear' risk of bias between the two reviews. According to the Cochrane handbook, the items blinding of outcome assessment and incomplete outcome data should be assessed for each outcome – we will follow this guidance. The qualitative assessment will be conducted independently by two epidemiologists. Any disagreements between reviewers were solved by discussion to reach consensus.

28. * Strategy for data synthesis.

Provide details of the planned synthesis including a rationale for the methods selected. This **must not be generic text** but should be **specific to your review** and describe how the proposed analysis will be applied to your data.

We will conduct a qualitative synthesis, organizing studies according to when addiction treatment was provided (e.g. before, during, after incarceration or in a continuum of treatment including at least two of those periods).. No meta-analysis will be performed. We anticipate high heterogeneity due to the inclusion of different populations (currently incarcerated and recent released persons), different treatments to treat opioid use disorder (e.g. methadone, buprenorphine, naltrexone) or to prevent opioid-related overdose (e.g. naloxone), and different study outcomes (e.g. relapse into opioid use, initiation and adherence to addiction treatment measured with different strategies, treatment side-effects, opioid-related mortality etc). We are facing an opioid-related overdose crisis, end people with opioid use disorders are over-represented in the criminal system. However, prison and jails often adopt an abstinence-only approach, seldom providing the gold standard treatment to incarcerated persons with opioid use disorders (opioid agonist treatment). It is our intent to contribute with this discussion and inform more adequate and ethical management of incarcerated persons struggling with opioid use disorders before, during and after incarceration.

29. * Analysis of subgroups or subsets.

State any planned investigation of 'subgroups'. Be clear and specific about which type of study or participant will be included in each group or covariate investigated. State the planned analytic approach.

It will not be conducted under this review

30. * Type and method of review.

Select the type of review and the review method from the lists below. Select the health area(s) of interest for your review.

Type of review

Cost effectiveness	No
Diagnostic	No
Epidemiologic	Yes
Individual patient data (IPD) meta-analysis	No
Intervention	Yes
Meta-analysis	No
Methodology	No
Narrative synthesis	Yes
Network meta-analysis	No
Pre-clinical	No
Prevention	No
Prognostic	No
Prospective meta-analysis (PMA)	No
Review of reviews	No
Service delivery	No
Synthesis of qualitative studies	No
Systematic review	Yes
Other	No

Health area of the review

Alcohol/substance misuse/abuse	Yes
Blood and immune system	No
Cancer	No
Cardiovascular	No
Care of the elderly	No
Child health	No
Complementary therapies	No

Crime and justice	No
Dental	No
Digestive system	No
Ear, nose and throat	No
Education	No
Endocrine and metabolic disorders	No
Eye disorders	No
General interest	No
Genetics	No
Health inequalities/health equity	No
Infections and infestations	No
International development	No
Mental health and behavioural conditions	No
Musculoskeletal	No
Neurological	No
Nursing	No
Obstetrics and gynaecology	No
Oral health	No
Palliative care	No
Perioperative care	No
Physiotherapy	No
Pregnancy and childbirth	No
Public health (including social determinants of health)	No
Rehabilitation	No
Respiratory disorders	No
Service delivery	No
Skin disorders	No

Social care	No
Surgery	No
Tropical Medicine	No
Urological	No
Wounds, injuries and accidents	No
Violence and abuse	No

31. Language.

Select each language individually to add it to the list below, use the bin icon to remove any added in error.

English

There is not an English language summary

32. * Country.

Select the country in which the review is being carried out from the drop down list. For multi-national collaborations select all the countries involved.

Canada

33. Other registration details.

Give the name of any organisation where the systematic review title or protocol is registered (such as with The Campbell Collaboration, or The Joanna Briggs Institute) together with any unique identification number assigned. (N.B. Registration details for Cochrane protocols will be automatically entered). If extracted data will be stored and made available through a repository such as the Systematic Review Data Repository (SRDR), details and a link should be included here. If none, leave blank.

34. Reference and/or URL for published protocol.

Give the citation and link for the published protocol, if there is one

No I do not make this file publicly available until the review is complete

35. Dissemination plans.

Give brief details of plans for communicating essential messages from the review to the appropriate audiences.

Open access scientific manuscripts

Do you intend to publish the review on completion?

Yes

36. Keywords.

Give words or phrases that best describe the review. Separate keywords with a semicolon or new line. Keywords will help users find the review in the Register (the words do not appear in the public record but are included in searches). Be as specific and precise as possible. Avoid acronyms and abbreviations unless these are in wide use.

opioid use disorder; correctional adult population; overdose

37. Details of any existing review of the same topic by the same authors.

Give details of earlier versions of the systematic review if an update of an existing review is being registered, including full bibliographic reference if possible.

38. * Current review status.

Review status should be updated when the review is completed and when it is published. For newregistrations the review must be Ongoing.

Review_Ongoing

39. Any additional information.

Provide any other information the review team feel is relevant to the registration of the review.

40. Details of final report/publication(s).

This field should be left empty until details of the completed review are available.
