# Supplementary material for: Opioid-related treatment, interventions, and outcomes among incarcerated persons: A systematic review
Source: PLoS Med. 2019 Dec 31;16(12):e1003002. doi: 10.1371/journal.pmed.1003002 (PMC6938347; doi:10.1371/journal.pmed.1003002)
Supplement: Attachment — Submitted filename: malta.pdf [file pmed.1003002.s005.pdf]

This paper reports a systematic review of studies of opioid related treatments in correctional populations.

I was asked for a statistical report and I interpret that to include all aspects of the design and conduct of the study.

Points of detail

Page 6 Is there any specific reason to start the search in 2008? Opioids have been the subject of use and mis–usefor longer than that.

Page 8 If authors were asked to supply extra data then I think that (a) we should be told which items we are not going to be able to find in the original publications (b) the people who supplied it should be acknowledged.

Page 9 How was it decided which of the duplicates to include?

Page 10 It is good to potentially include material not in English but I wonder whether the choice of bibliographic data bases is optimal for that purpose. Various empirical studies have indicated that the main bibliographic databases have varied and incomplete coverage of material not in English (Pilkington et al., 2005), and have limited coverage of material from low and middle–income country journals (Kieling et al., 2009; Syed Sheriff et al., 2008). There are some other databases sponsored by WHO.

Page 23 Why does the caption say selected? How were they selected and for what purpose?

Points of more substance

Presentation of results

I found the whole results section rather forbidding. I wonder whether presenting the studies in one table like this is ideal? Why not split it into multiple tables corresponding to the same sections into which the text is currently split? Then the reader does not have so many pages to jump to cross refer text to numbers? This might mean that the text would need to replicate less of the tables too.

Effect sizes

Many of the studies are summarised in the table and text purely as p–values. Is this because the primary studies only give that information and the present authors have not been able to obtain it or calculate it from the primary articles?

Meta–analysis

The authors have not carried out a meta–analysis. I think we would benefit from knowing, possibly in the methods section, why they made that decision. I can see a whole host of possibilities. Of course there is always the last resort of combining p–values using Fisher's method, Stouffer's method, or one of the other options. Loughin (2004) compares some of the methods.

Summary

Mostly comments about clarification and expansion.

Michael Dewey

References

C Kieling, H Herrman, V Patel, and J J Mari. Indexation of psychiatric journals from low– and middle–income countries: a survey and a case study. *World Psychiatry*, 8:40–44, 2009.

T M Loughin. A systematic comparison of methods for combining p–values from independent tests. *Computation Statistics and Data Analysis*, 47: 467–485, 2004.

K Pilkington, A Boshnakova, M Clarke, and J Richardson. 'No language restrictions' in database searches: what does this really mean? *The Journal of Alternative and Complementary Medicine*, 11:205–207, 2005.

R J Syed Sheriff, C E Adams, P Tharyan, M Jayaram, L Duley, and the PRACTIHC Mental Health Group. Randomised trials relevant to mental health conducted in low and middle–income countries: a survey. *BMC Psychiatry*, 8(69), 2008.
