# Supplementary material for: Opioid-related treatment, interventions, and outcomes among incarcerated persons: A systematic review
Source: PLoS Med. 2019 Dec 31;16(12):e1003002. doi: 10.1371/journal.pmed.1003002 (PMC6938347; doi:10.1371/journal.pmed.1003002)
Supplement: Attachment — Submitted filename: Rebuttal Letter.docx [file pmed.1003002.s006.docx]

PMEDICINE-D-19-02187

"Opioid-Related Treatment, Interventions and Outcomes among Correctional Populations: A Systematic Review"

Plos Medicine

Dear Dr. Stone and Dr. Turner,

We would like to thank the editors and reviewers for their comments and suggestions to improve our manuscript. We addressed all reviewers' and editors' comments, please see below a point-by-point response to the review.

Associate Editor Comments:

**1. Please include in an Appendix document a list of the 38 studies deemed ineligible for reasons of aim/outcome/intervention focus.**

R: Appendix included. The vast majority of those studies included criminal justice involved substance users who were either on parole or probation.

**2. The first paragraph provides a reasonable summary of the evidence about health among incarcerated persons. But at least one additional point of the manuscript (or a point that the manuscript _should_ make) is that people with OUDs who have recently been incarcerated are a uniquely vulnerable population. Detoxification without linkage to OAT increases overdose risk substantially (Merrall EL et al, Addiction. 2010;105(9):1545-54. Binswanger IA et al, Ann Intern Med. 2013;159:592-600), but neither OAT nor linkage are commonly provided (Aronowitz SV et al, J Correct Health Care. 2016;22(2):98-108. Friedmann PD et al, Subst Abus. 2012;33(1):9-18).**

R: Paragraph reorganized, relevant references included.

**3. At some point in the introduction or discussion, the authors should make the point that OAT has been found to reduce mortality in non-criminal justice involved populations (eg., Larochelle MR, Ann Intern Med. 2018;169(3):137-45. Sordo L et al, BMJ. 2017;357:j1550. Ma J et al, Mol Psychiatry, in press, doi:10.1038/s41380-018-0094-5). Benefits on a wide range of other non-mortality outcomes have also been described, including HIV transmission risk behavior. The discussion in particular would be enhanced if the authors contrasted their findings with studies of non-criminal justice involved populations. It is well known that OAT reduces mortality. It should not come as a surprise that OAT reduces mortality among those who have been incarcerated or recently released. ** It would be useful if the authors could contextualize their findings in light of this other literature-- are the effect sizes observed among incarcerated/released persons larger or smaller than in non-incarcerated populations (and why)? ****

R: We thank the editor for this suggestion, and an updated discussion is included comparing the impact of OAT on mortality rate of incarcerated vs. never incarcerated persons diagnosed with OUD.

**Please note that I am _not_ requiring that the authors cite the specific manuscripts listed above. Many are big papers in the field, but if the authors have alternative citations to support these claims, those could certainly be defended.**

R: We included relevant and appropriated references in the introduction and discussion of our revised manuscript.

**4. R3 requests that the phrase "during incarceration" be changed, but in my opinion it appears that there are several instances in the manuscript where the phrase "during incarceration" is appropriate and does not need to be changed.**

R: We revised the manuscript and included a better explanation of when (1) opioid use and/or (2) opioid agonist treatment was measured and identified ‘during incarceration’, therefore acknowledging both the editor and R3 suggestions.

**5. I am in agreement with R3 that the Discussion--particularly paragraphs 2-4--is better described as a rehashing of the Results rather than a summative synthesis of the findings. Please rewrite as appropriate, synthesizing the findings across studies/across interventions/across time points in the criminal justice system cascade.**

R: We thoroughly revised the discussion, following the editor and reviewers recommendations.

**Editorial comments:**

**Title – please provide country setting Abstract – related to above point, you open the abstract by mentioning the US specifically. Please state if the studies you select are based on US cohorts. I see some are from NSW, so perhaps the opening of the abstract could have a more global perspective?**

R: We revised the abstract to reflect the multi-country nature of our review.

**Abstract – please avoid a list like approach using numbers to list points and please provide a sentence of limitations as the final sentence of the Methods and Findings section of the abstract. Finally, please avoid using causal language, as this is a systematic review (strong evidence)**

R: We revised the abstract accordingly.

**At this stage, we ask that you include a short, non-technical Author Summary of your research to make findings accessible to a wide audience that includes both scientists and non-scientists. The Author Summary should immediately follow the Abstract in your revised manuscript. This text is subject to editorial change and should be distinct from the scientific abstract. Please see our author guidelines for more information:** <https://journals.plos.org/plosmedicine/s/revising-your-manuscript#loc-author-summary>

R: Author Summary included.

**There is perhaps some kind of formatting issue in the methods section ((i.e. exp Opiate Substitution Treatment/, buprenorphine$, methadone$, naltrexone$, naloxone$) among incarcerated and post-incarceration populations (i.e. jail$, prison$, offender$, probat$, felon$, detention$, imprison$, postincarcerat$) who use opioids (i.e. exp Opioid-Related Disorders/, opiate$, opioid$)) – please correct here and elsewhere, as needed.**

R: We apologize; the $ is for truncation and was utilized on the bibliographic search. The paragraph was revised and includes only the actual keywords utilized, The full search strategy is available son Appendix XYZ

**Please provide 95% Cis as well as p values for all quantifiable data.**

R: 95% CI, p-values and SD were included when available.

**Please use the "Vancouver" style for reference formatting, and see our website for other reference guidelines** <https://journals.plos.org/plosmedicine/s/submission-guidelines#loc-references>

R: References reviewed.

**Thank you for providing the PRISMA checklist – please use sections and paragraphs instead of page numbers, as these can change on revisions or publication.**

R: PRISMA checklist reviewed.

**Comments from the reviewers:**

**Reviewer #1: See attachment**

**Reviewer #2: This is a systematic review of peer reviewed literature in the past decade (2008-2018) of opioid use interventions delivered during and post incarceration/release among adult correctional populations. The 45 selected studies from the 2,323 articles reviewed provide strong support for the positive impact of opioid related interventions during and post release from incarceration. The selections of the studies and the descriptions of the studies are appropriate, as are the conclusions.**

**It is important to note that incarceration and criminal justice involvement, in many countries, especially in the US, is part of the natural history of this disease. The introduction describes the "correctional population" as if they are a different species almost, and clearly there are differences, but it is important to keep in mind that this is the same disease.**

R: We agree, the introduction and discussion were revised accordingly.

**It would be helpful to include in one of the tables whether the study was done in a jail, a prison population or combined, or other population. There should also be some brief discussion of the differences between interventions in jails (much larger numbers and shorter times incarcerated) and prisons (smaller numbers, more time to work with people).**

R: We included an additional column on Table 1, identifying the setting where each study was conducted (prison, jail, before/during/after prison). An additional discussion comparing interventions conducted in jails vs. prisons was included.

**p-5-6, sentence about linkage points should include probation and parole (as part of the "etc").**

R: Included.

**p-8, why did the authors decide to exclude drug courts and probation and parole? I agree with that choice but that should be described. [also, the authors should consider doing 2 more systematic reviews- one on drug courts and one on probation and parole- those would also be substantial contributions to the field]**

R: We included one brief paragraph explaining this methodological decision. We agree that more systematized information is needed about the availability, effectiveness and impact of OAT among parolees and probationers with OUD. However this additional study should be conducted within another systematic analysis, in order to address the specificities of this population and the impact of being in the community on both OUD, opioid use relapse and access/adherence to OAT. We appreciate the reviewer suggestion.

**p-8, line 4, when "initiated" is used, does that include people who were continued on medications during incarceration? I believe it does or should, and just needs to be explicit.**

R: We clarified when incarcerated persons started OAT in prison, and when they actually continued the treatment started before incarceration.

**p-8, lines 8-10, should also explain why all of these studies are excluded**

R: We included one sentence to better explain the focus of our systematic review, and underlying reasons for our eligibility criteria.

**p-32, line 19 uses the term "offenders". Although that term is frequently used, it should be avoided as 1) it is further stigmatizing of an already severely stigmatized population, and 2) it is not always accurate, as many people are erroneously incarcerated even though they have not committed an offense.**

**Person first language is better. IE an incarcerated person.**

R: We thank the reviewer for the careful reading. All sentences were revised to avoid any stigmatizing term (e.g. offenders, inmates etc). Study participants are now referred as ‘incarcerated persons’ throughout the manuscript and tables.

**p-37 line 16 typo "to into"**

R: Reviewed.

**p-42, line 5, avoid the term "offenders"**

R: “Offenders” were replaced by “incarcerated persons’.

**p-42, line 10, what is "criminal recidivism"? is that re-incarceration? re-incarceration based upon new charges, a new conviction, or does it include also people incarcerated for parole or probation violations?**

R: We replaced ‘criminal recidivism’ by ‘re-incarceration’. When additional information was available, we included the reasons for re-incarceration.

**p-44, lines 5, 7, and 8, these percentages (and probably most in the article) should be rounded to whole numbers. The decimal points imply a degree of accuracy that the data certainly does not support and they are distracting.**

R: Revised.

**Page 48, line 2- again, I would suggest a separate paper reviewing treatment of opioid use disorder in probation and parole because the numbers of individuals is much larger.**

R: We will consider the reviewer suggestion, if this study has not been done yet.

**p-48, line 13, "post-continues" is a typo**

R: Revised.

**Reviewer #3: This is an important paper on a very important topic. However, the paper needs to be strengthened before it is ready for publication:**

**1) The terms used throughout are not consistently used or defined. A few examples: a) the current paradigm we are in is an overdose crisis, not an opioid crisis. Would change language throughout to emphasize this; b) suggest using person first language throughout rather than inmate, etc.; c) Often the authors talk about opioid use "during incarceration", but they don't mean this--they mean "among people with criminal justice experience. This should be changed throughout; d) they also often refer to "opioid use interventions", but this is not comprehensive enough. They mean opioid-related interventions more broadly because many interventions (for example, naloxone distribution) aren't targeting opioid use, but instead preventing overdose; e) finally, suggest using one term to refer to the criminal justice system throughout rather than incarceration, corrections, etc.**

R: Following the reviewer suggestions, we replaced (1) opioid crisis by opioid-related overdose crisis; (2) inmate/ offenders were replaced by ‘incarcerated persons’ to avoid any stigmatizing term; (3) every intervention was briefly explained to avoid confusions between strategies conducted during incarceration and those implemented after prison release. Additional information was included to differentiate interventions utilizing opioid agonist therapy (OAT) and opioid overdose prevention. The term ‘criminal justice system’ was utilized when adequate, while when we were referring to a specific environment (e.g. prison), we maintained the specificity.

**2) In the methods they ought to explain why they looked at the years 2008-2018 and why they included only folks within 90 days of release.**

R: We included a better explanation on the methods section. Briefly, there are other similar systematic reviews available, and our major goal was to update the available information with more recent studies, including in our study OAT innitiated before, during and after incarceration. See, for instance:

Hedrich D, Alves P, Farrell M, Stöver H, Møller L, Mayet S. The effectiveness of opioid maintenance treatment in prison settings: a systematic review. Addiction. 2012 Mar;107(3):501-17.

Our review included studies conducted with incarcerated persons or those recently released into the community (≤ 90 days post-incarceration/release). The main reason to include person recent released is to evaluate the impact of OAT (before, during or immediately after release) on post-release opioid-related overdose risk. Post-release opioid-related overdose mortality is a leading cause of death among people released from jails or prisons, and this increased mortality is often identified within the first 1-3 months after release. See, for instance:

Binswanger IA, Blatchford PJ, Mueller SR, Stern MF. Mortality after prison release: opioid overdose and other causes of death, risk factors, and time trends from 1999 to 2009. Ann Intern Med. 2013 Nov 5;159(9):592-600

Joudrey PJ, Khan MR, Wang EA, Scheidell JD, Edelman EJ, McInnes DK, Fox AD. A conceptual model for understanding post-release opioid-related overdose risk. Addict Sci Clin Pract. 2019 Apr 15;14(1):17

We included an additional explanation in our methods section.

**3) On page 5 when talking about the criminal justice system continuum, they should refer to and/or be familiar with Brinkley-Rubinstein (2018) Criminal Justice Continuum for Opioid Users at Risk of Overdose:** <https://www.ncbi.nlm.nih.gov/pubmed/29544869>

R: The highly important concept of a “CJ Continuum of Care for Opioid Users at Risk of Overdose”, as introduced by Dr. Lauren Brinkley-Rubinstein and colleagues, was pivotal to guide our study. A more complete reference for this seminal concept was included.

**4) When describing the particular studies in the result it is important to be very precise. For example, in the implant naltrexone section, the authors refer to how those with the implant were more likely to uptake treatment in the community, but it is unclear at what time point (one month, six months??) and whether they mean naltrexone specifically or any MAT. This precision should be applied to all text when referring to specific studies.**

R: We revised the results section carefully, to include a more complete description of each study and its major outcomes.

**5) The discussion, in many ways, rehashes the results instead of expanding them and making recommendations for how they might be used to expand MAT program implementation. Suggest, adding more in this vein.**

R: We completely revised our discussion section, in order to address this key public health concern: why, after decades of studies and well-know OAT efficacy, incarcerated persons with OUD are still denied treatment? It is our hope that this study will contribute somehow to change this unacceptable human rights violation.

**6) Finally, perhaps the biggest flaw of this paper, is the fact that there is no attention paid to country-specific context. For example the implantable naltrexone study (which there's only one of by the way) comes out of Norway, which has both a very, very different criminal justice landscape than the US and also more affordable access to healthcare. So, these results cannot be broadly applied to the US context--the authors must take the US context into consideration especially since the US overdose epidemic is the motivation for the paper in the first place. Therefore, for this paper to be acceptable for publication there must be some contextual overlay of the US criminal justice system and the lack of access to MAT in both corrections-based and community-based settings in the US.**

R: The US context was not the motivation of our systematic review. However we agree with the reviewer that the US context of (1) opioid-related overdose crisis and (2) incarceration rate - the highest in the world, are highly important. Therefore we thoroughly revised our discussion, highlighting the magnitude and importance of the opioid-related overdose crisis in the US, and the importance to scale up OAT among this highly vulnerable population: people with OUD who are involved in the criminal system.

**R4**

**This paper reports a systematic review of studies of opioid related treatments in correctional populations. I was asked for a statistical report and I interpret that to include all aspects of the design and conduct of the study.**

**Points of detail**

**Page 6 Is there any specific reason to start the search in 2008? Opioids have been the subject of use and misused for longer than that.**

R: We included a better explanation on the methods section. Briefly, there are other similar systematic reviews available that thoroughly evaluated studies conducted before 2008 and our major goal was to update the available information with more recent studies, including in our study OAT innitiated before, during and after incarceration. See, for instance:

Hedrich D, Alves P, Farrell M, Stöver H, Møller L, Mayet S. The effectiveness of opioid maintenance treatment in prison settings: a systematic review. Addiction. 2012 Mar;107(3):501-17.

**Page 8 If authors were asked to supply extra data then I think that (a) we should be told which items we are not going to be able to find in the original publications (b) the people who supplied it should be acknowledged.**

R: We conducted a thorough evaluation of all data available in each selected manuscript, and although our research team contacted several authors, no additional data or study clarification was sent or included in our systematic review. Therefore we opted to omit this information.

**Page 9 How was it decided which of the duplicates to include?**

R: When duplicates were identified, the study with more relevant data related to the impact of OAT was included. For instance, if a research group evaluated the impact on in-prison OTA on heroin relapse, but later evaluated the impact of in-prison OAT on heroin relapse, OAT adherence and re-incarceration, this study was included.

**Page 10 It is good to potentially include material not in English but I wonder whether the choice of bibliographic data bases is optimal for that purpose. Various empirical studies have indicated that the main bibliographic databases have varied and incomplete coverage of material not in English (Pilkington et al., 2005), and have limited coverage of material from low and middle-income country journals (Kieling et al., 2009; Syed Sheri_ et al., 2008). There are some other databases sponsored**

**by WHO.**

R:

The Cochrane Collaboration recommends searching without language restrictions to avoid language bias. It also suggests searching regional databases when relevant. We included, for instance, the US National Criminal Justice Reference Service database (NCJRS), Embase (key database for European peer reviewed journals). We didn’t have any language limit in our search, and the majority of included databases index foreign language journals. Embase is especially valuable for identifying relevant research from non-North American journals. Over 49 percent of the journals indexed by Embase are European, 82 percent of the titles are unique to Embase and not indexed by Medline are published outside North America. To search for LMIC studies, we conducted additional searchers in the following databases: African Index Medicus, Africa Portal Digital Library, Index Medicus for the Southeast Asia Region, IndMed, and Latin American and Caribbean Health Science Literature Database (LILACS). However, no additional and eligible study was identified. This second search is also described on the revised manuscript, and we thank the reviewer for this important suggestion.

**Page 23 Why does the caption say selected? How were they selected and for what purpose?**

R: There was no study selection, all eligible studies were included on the tables. We changed the table title to avoid any confusion.

**Points of more substance**

**Presentation of results**

**I found the whole results section rather forbidding. I wonder whether presenting the studies in one table like this is ideal? Why not split it into multiple tables corresponding to the same sections into which the text is currently split? Then the reader does not have so many pages to jump to cross refer text to numbers? This might mean that the text would need to replicate less of the tables too.**

R: We thank the reviewer for this suggestion. The results are now presented in 4 tables, corresponding to specific sub-headings.

**Effect sizes**

**Many of the studies are summarised in the table and text purely as p{values. Is this because the primary studies only give that information and the present authors have not been able to obtain it or calculate it from the primary articles?**

R: We reviewed all selected studies, and included additional information for all studies (e.g. 95% CI, SR, SE)

**Meta-analysis**

**The authors have not carried out a meta-analysis. I think we would benefit from knowing, possibly in the methods section, why they made that decision. I can see a whole host of possibilities. Of course there is always the last resort of combining p-values using Fisher's method, Stouffer's method, or one of the other options. Loughin (2004) compares some of the methods.**

R: We decided not to conduct a meta-analysis, and included one additional paragraph explaining our methodological decision. In brief, our systematic review included 45 studies with a high heterogeneity: different populations (incarcerated and non-incarcerated persons), different interventions (methadone, buprenorphine, naltrexone, naloxone), different outcomes (relapse into opioid use, initiation or adherence to OAT, re-incarceration, treatment side-effects, opioid-related mortality etc). We attempted to combine studies within small groups according to the intervention (buprenorphine, methadone, naltrexone and naloxone), but the outcomes utilized were different, or methods/timeframe utilized to measure similar outcomes were diverse. Therefore, after attempting to conduct meta-analysis of small groups we realized that the average effect across studies was meaningless.

A smaller meta-analysis with similar objective, including only RCT studies was recently published and is cited in our discussion:

Moore KE, Roberts W, Reid HH, Smith KMZ, Oberleitner LMS, McKee SA. Effectiveness of medication assisted treatment for opioid use in prison and jail settings: A meta-analysis and systematic review. J Subst Abuse Treat. 2019 Apr;99:32-43.

**Summary**

**Mostly comments about clarification and expansion.**

We would like to thank all reviewers and editor for the careful evaluation of our manuscript, highly important critiques and suggestions.
